# Evaluating potential predictors of weight loss response to liraglutide in adolescents with obesity: A post hoc analysis of the randomized, placebo-controlled SCALE Teens trial

**DOI:** 10.1111/ijpo.13061

**Published:** 2023-06-01

**Authors:** Megan O. Bensignor, Carolyn T. Bramante, Eric M. Bomberg, Claudia K. Fox, Paula M. Hale, Aaron S. Kelly, Rashmi Mamadi, Nandana Prabhu, Nina M. Harder-Lauridsen, Amy C. Gross

**Affiliations:** 1 Department of Pediatrics, Center for Pediatric Obesity Medicine, University of Minnesota, Minneapolis, Minnesota, USA; 2 Clinical Development, Medical & Regulatory Affairs, Novo Nordisk Inc., Plainsboro, New Jersey, USA; 3 Global Medical Affairs, Novo Nordisk, Bangalore, India; 4 Biostatistics, Novo Nordisk, Bangalore, India; 5 Medical and Science, Novo Nordisk A/S, Søborg, Denmark

**Keywords:** anti-agents, glucagon-like peptide-1 receptor agonists, liraglutide, obesity, obesity, paediatric obesity, weight management

## Abstract

**Background:**

As childhood obesity prevalence increases, determining which patients respond to anti-obesity medications would strengthen personalized approaches to obesity treatment. In the SCALE Teens trial among pubertal adolescents with obesity (NCT02918279), liraglutide 3.0 mg (or maximum tolerated dose) significantly reduced body mass index (BMI) standard deviation score on average versus placebo. That said, liraglutide effects on BMI reduction varied greatly among adolescents, similar to adults.

**Objectives:**

To identify post hoc characteristics predictive of achieving ≥5% and ≥10% BMI reductions at 56 weeks with liraglutide versus placebo in adolescents from the SCALE Teens trial.

**Methods:**

Logistic regression analysis was performed in 251 adolescents treated with liraglutide (*n* = 125) or placebo (*n* = 126) for 56 weeks. Baseline characteristics (selected a priori) included sex, race, ethnicity, age, Tanner (pubertal) stage, glycemic status (hyperglycemia [type 2 diabetes/prediabetes] vs. normoglycemia), obesity category (Class II/III vs. I), severity of depression symptoms (Patient Health Questionnaire-9), and weight variability (weight fluctuations over time). The effects of early responder status (≥4% BMI reduction at week 16) on week 56 response were assessed using descriptive statistics.

**Results:**

Baseline characteristics did not affect achievement of ≥5% and ≥10% BMI reductions at week 56 in adolescents treated with liraglutide. Further, there was no association between weight variability and BMI reduction. Early liraglutide responders appeared to have greater BMI and body weight reductions at week 56 compared with early non-responders.

**Conclusions:**

This secondary analysis suggests that adolescents with obesity may experience significant BMI reductions after 56 weeks of liraglutide treatment, regardless of their sex, race, ethnicity, age, pubertal stage, glycemic status, obesity category, severity of depression symptoms, or weight variability. Early response may predict greater week 56 response.

## 1 | INTRODUCTION

The worldwide prevalence of overweight and obesity in children and adolescents has increased ~4.5-fold over the last 40 years, from 4% in 1975 to above 18% in 2016.^1^ Obesity is a multifactorial, chronic, and relapsing disease with high variation in individual treatment response.^2–5^ While lifestyle therapy is the cornerstone of treatment, several studies suggest that only 2%–15% of adolescents with severe obesity (paediatric Class II [body mass index (BMI) 120%–139% of the 95th percentile]/Class III [BMI ≥140% the 95th percentile]^6^) show clinically significant and durable reductions in weight or BMI with this approach alone.^7^ To date, few anti-obesity medications (AOMs) indicated for chronic weight management in adolescents have proven to be safe and effective, and/or have been approved globally.^8^ Liraglutide is a glucagon-like peptide-1 receptor agonist (GLP-1RA) first approved for use in adolescents with obesity in the United States in 2020, followed by Europe in 2021 (where it was the first AOM approved for adolescents). It is indicated for obesity management at daily doses of up to 3.0 mg in adolescents aged ≥12 years, as an adjunct to lifestyle therapy.^9,10^

A clinically and significantly greater BMI standard deviation score (SDS) reduction was achieved with liraglutide 3.0 mg compared with placebo in pubertal adolescents with obesity in the Satiety and Clinical Adipose Liraglutide Evidence (SCALE) Teens trial, with an estimated treatment difference of −0.22 (95% confidence interval: −0.37, −0.08).^11^ Variability in liraglutide response was observed in the SCALE Teens trial, with BMI reductions of ≥5% and ≥10% reported in 43.3% and 26.1%, respectively, of participants in the liraglutide group (vs. 18.7% and 8.1%, respectively, with placebo).^11^ Similar findings were observed in the adult SCALE Obesity and Prediabetes trial, in which 63.2% and 33.1% of participants in the liraglutide group lost ≥5% and ≥10%, respectively, of their body weight (vs. 27.1% and 10.6%, respectively, with placebo).^12^

It is well-known that the magnitude of response to pharmacologic and non-pharmacologic weight loss treatments can vary considerably across individuals.^5,13^ For AOMs, potential predictors of response in both adults and adolescents include sex, age, baseline body weight, baseline BMI, baseline depressive symptoms, baseline blood glucose levels, and weight variability (the magnitude of individual body weight fluctuations recorded over a period of time).^5,13–17^ In addition, early response to treatment (commonly assessed after 12 weeks of intervention) has repeatedly been shown to predict later response to weight loss interventions in adults and adolescents.^5,10,18–22^

Determining which adolescent patients are likely to respond to AOMs could contribute to more personalized approaches for obesity treatment.^23,24^ Specifically, identifying predictors of response could allow for reductions in unnecessary exposure to medications and inefficient use of healthcare resources. Therefore, the purpose of this post hoc analysis of the SCALE Teens trial was to identify potential characteristics that may predict a clinically relevant response in adolescents treated with liraglutide 3.0 mg compared with placebo.

## 2 | METHODS

### 2.1 | Participants and trial design

**FIGURE 1:**
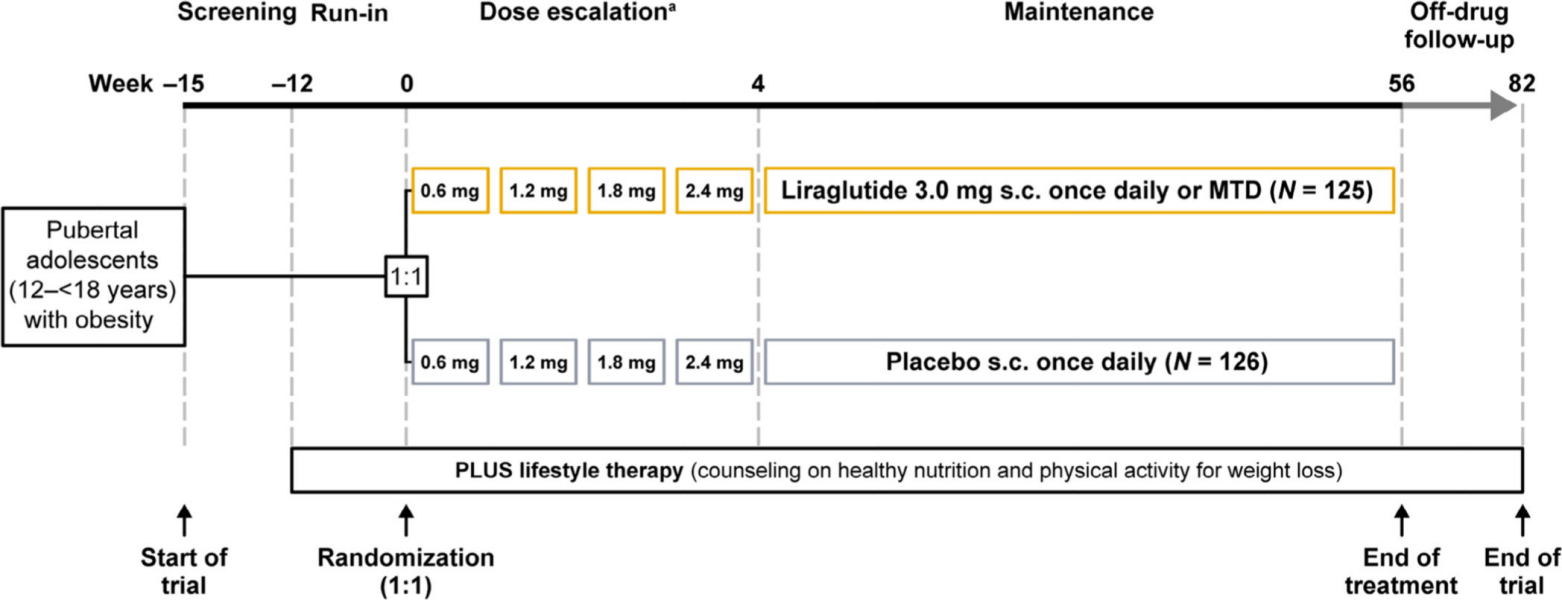
Study design. The SCALE Teens trial included a 12-week lifestyle therapy-only run-in period, a 56-week treatment period (during which participants were randomized 1:1 to receive liraglutide or placebo, and which consisted of a 4-week dose escalation and 52-week maintenance phases), and a 26-week off-treatment lifestyle therapy-only follow-up period. Participants received lifestyle therapy throughout the entire trial. MTD, maximum tolerated dose; s.c., subcutaneous; SCALE, Satiety and Clinical Adipose Liraglutide Evidence. ^a^Dose escalation could be prolonged up to 8 weeks if required.

The design, methods (including protocol and statistical analysis plan), trial population, and results of the SCALE Teens trial (ClinicalTrials.gov: NCT02918279) have been previously published.^11^ Briefly, the SCALE Teens trial evaluated the safety and efficacy of liraglutide 3.0 mg in 251 adolescents with obesity, with or without type 2 diabetes (T2D), and with insufficient response to lifestyle therapy, who were randomized 1:1 to liraglutide or placebo. The trial was conducted at 32 sites in five countries (Belgium, Mexico, Russia, Sweden, and the United States) and included a 12-week lifestyle therapy-only run-in period, 56-week treatment period, and 26-week lifestyle therapy-only follow-up period (Figure 1). Lifestyle therapy was provided to all participants from screening to the end of the trial. Liraglutide was initiated at 0.6 mg daily and escalated weekly by 0.6 mg until the target dose (3.0 mg daily) or maximum tolerated dose was reached. The primary endpoint of the trial was change in BMI SDS from baseline to week 56. Secondary endpoints included the percentages of participants who had reductions in BMI of ≥5% and ≥10% at week 56.^11^

Eligible participants were pubertal adolescents aged 12 to <18 years with a BMI corresponding to ≥30 kg/m^2^ for adults by international cut-off points.^25^ Adolescents with T2D were eligible, but those taking anti-diabetic treatment (other than metformin) and those with type 1 diabetes were excluded. Tanner staging was conducted by site staff trained in pubertal assessment in accordance with stages 1–5, including assessment of male testicular volume using an orchidometer. Once participants reached Tanner stage 5, as judged by the investigator, Tanner staging was no longer required.^11^

The trial was conducted according to Good Clinical Practice guidelines and the Declaration of Helsinki. Independent national ethics committees or institutional review boards at each site approved the trial protocol and any subsequent amendments. Safety of the participants and evaluation of the benefit–risk balance was also overseen on a global trial level by an independent external data monitoring committee. Written informed consent was obtained from all parents or legally acceptable representatives and informed assent was obtained from all participants before the initiation of any trial-related procedures.^11^

### 2.2 | Statistical analysis

This post hoc analysis of the SCALE Teens trial was performed on the full analysis set, which included all randomized participants receiving at least one dose of trial product and who had any post-randomization data.

Logistic regression analyses were performed using baseline characteristics selected a priori to investigate whether they were predictive of achieving ≥5% and ≥10% BMI reductions after 56 weeks. Odds ratios of achieving these BMI percentage reductions were determined by the regression analyses. The pre-specified baseline characteristics analysed included biologic sex, race (white vs. non-white), ethnicity (non-Hispanic/Latino vs. Hispanic/Latino; ethnicity was self-assigned in accordance with nationally agreed guidelines [US Food and Drug Administration]), age group (12–14 vs. 15–17 years), Tanner stage (2–3 vs. 4–5), glycemic status (hyperglycemia [T2D or prediabetes] vs. normoglycemia), obesity category (Class II/III [severe] obesity [defined as either a BMI ≥120% of the 95th percentile or an absolute BMI ≥35 kg/m^2^, whichever was lower]^6^ vs. Class I obesity [defined as a BMI ≥95th percentile to <120% of the 95th percentile or an absolute BMI <35 kg/m^2^] measured on Centers for Disease Control and Prevention age- and sex-specific growth charts),^26^ and Patient Health Questionnaire-9 score (<5 vs. ≥5 to <15, corresponding to no or few symptoms of depression vs. symptoms of mild or moderate depression).^27^

Analyses used a jump to reference multiple (×100) imputation approach, where missing observations were imputed from the completed placebo group. Responses at week 56 were analysed using a logistic regression model according to the intention-to-treat principle, with sex and region (Europe and North America) as fixed effects, and baseline BMI and age as covariates. In addition, as randomization was stratified by Tanner stage and baseline glycemic status, these variables, as well as an interaction term between them, were included as fixed effects in the regression model.^28,29^

An early responder analysis was performed, using a definition for early response that was based on the stopping rules specified in the U.S. Food and Drug Administration (FDA) and European Medicines Agency (EMA) liraglutide prescribing information and the associated analysis in adults. Specifically, early responders were participants who achieved ≥4% BMI reduction at week 16 (4 weeks of dose escalation plus 12 weeks at the maintenance dose), whereas early non-responders did not.^9,10,20^ Achievement of ≥5% and ≥10% BMI reductions and mean changes in BMI and body weight at week 56 were assessed according to early response in each treatment group, among participants who had BMI measurements at baseline and week 16 (early response status for the change in body weight was based on ≥4% weight loss at week 16). The positive predictive value (proportions of early responders who achieved ≥5% and ≥10% BMI reductions at week 56) and negative predictive value (proportions of early non-responders who achieved <5% and <10% BMI reduction at week 56) were used to evaluate early response as a predictor of week 56 response. Of note, logistic regression analyses could not be performed because of the small number of participants in some of the groups.

A regression model was used to assess the linear relationship between change from baseline in BMI at week 56 and weight variability. Weight variability was calculated as the root mean square error around each participant’s regression line, using data from pre-randomization, lifestyle-only (weeks −12, −8, and −4), and baseline (week 0) visits. Association between BMI reduction at week 56 and weight variability during the lifestyle therapy-only run-in period was assessed using a scatter plot. The linear relationship between the change in BMI from baseline to week 56 was assessed using a multiple linear regression model fitted using baseline characteristics and weight variability.

## 3 | RESULTS

Of the 299 participants initially screened in the SCALE Teens trial, 251 were randomized to either liraglutide (*n* = 125) or placebo (*n* = 126).^11^ All randomized participants were included in this post hoc analysis (full analysis set). Baseline characteristics of the participants are shown in Table 1 (data for some reported parameters have previously been published^11^) and were well balanced between treatment groups. There were no notable differences in baseline characteristics between the early responders and early non-responders in each treatment group (Table S1).

**FIGURE 2:**
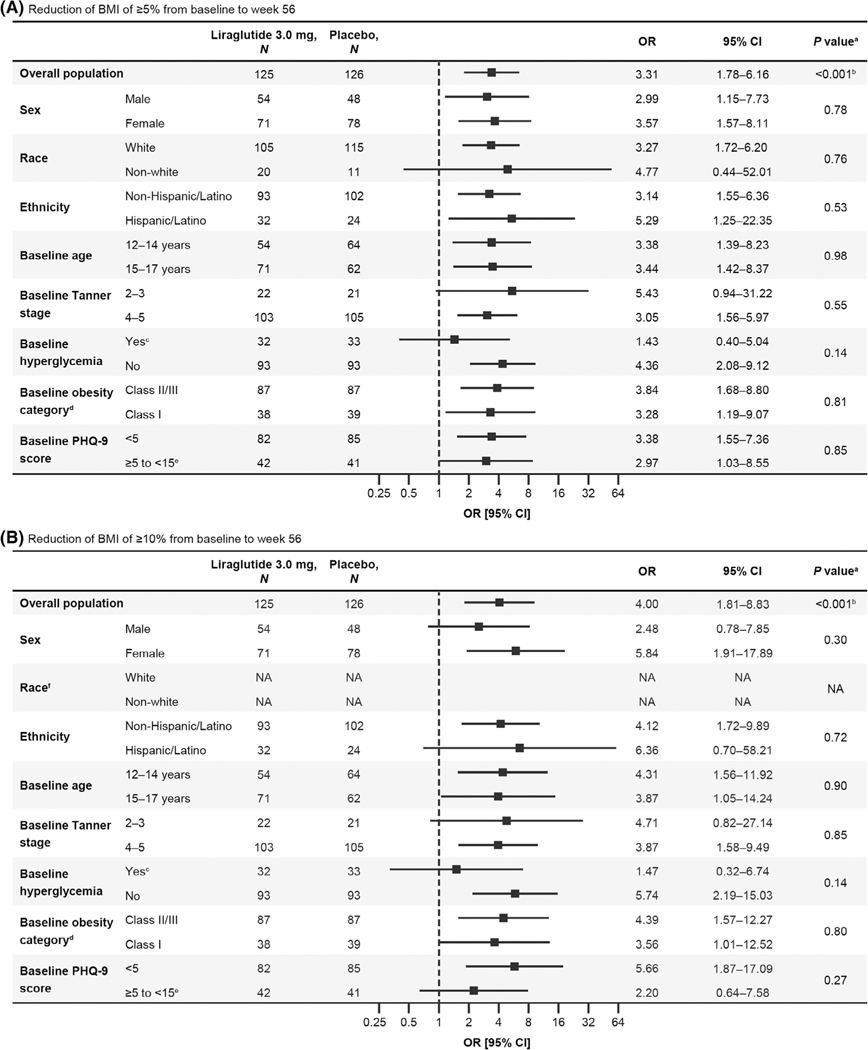
Subgroup analyses for reduction of BMI of (A) ≥5% and (B) ≥10% from baseline to week 56 for participants randomized to liraglutide versus placebo. Error bars are 95% CI. BMI, body mass index; CDC, Centers for Disease Control and Prevention; CI, confidence interval; NA, not available; OR, odds ratio; PHQ-9, Patient Health Questionnaire-9 score. ^a^The *p* value for treatment difference by subgroup interaction unless stated otherwise. ^b^The *p* value for treatment difference. ^c^Prediabetes (fasting plasma glucose 100 to ≤125 mg/dL [5.6 to ≤6.9 mmol/L] or a glycated haemoglobin level 5.7% to ≤6.4%) or type 2 diabetes (fasting plasma glucose ≥126 mg/dL [≥7.0 mmol/L] and/or a glycated haemoglobin level ≥6.5%). ^d^Class II/III (severe) obesity (BMI ≥120% of the 95th percentile and/or absolute BMI ≥35 kg/m^2^, whichever was lower)^6^ or Class I obesity (≥95th percentile to <120% of the 95th percentile or absolute BMI <35 kg/m^2^) defined by CDC age- and sex-specific growth charts.^26 e^Participants with PHQ-9 score ≥15 (indicating symptoms of severe depression) at screening were excluded from the trial. ^f^Analyses for race were not performed for a ≥10% reduction in BMI. In the model for which the predictor was race, no participants in the placebo group achieved this outcome between baseline and week 56; therefore, analyses could not be completed.

As shown in Figure 2, the odds of achieving BMI reductions of ≥5% or ≥10% from baseline to week 56 were greater among liraglutide-treated participants compared with those receiving placebo. There were no significant differences in odds ratios between subgroups in terms of sex (male vs. female, ≥5%: *p* = 0.78; ≥10%: *p* = 0.30), race (white vs. non-white, ≥5%: *p* = 0.76; odds ratio not available for ≥10% due to small number of participants), ethnicity (non-Hispanic/Latino vs. Hispanic/Latino, ≥5%: *p* = 0.53; ≥10%: *p* = 0.72), age (12–14 vs. 15–17 years, ≥5%: *p* = 0.98; ≥10%: *p* = 0.90), Tanner stage (2–3 vs. 4–5, ≥5%: *p* = 0.55; ≥10%: *p* = 0.85), baseline hyperglycemia (T2D or prediabetes vs. normoglycemia, ≥5%: *p* = 0.14; ≥10%: *p* = 0.14), obesity category (Class II/III vs. Class I, ≥5%: *p* = 0.81; ≥10%: *p* = 0.80), or severity of depression symptoms (Patient Health Questionnaire-9 score <5 vs. ≥5, ≥5%: *p* = 0.85; ≥10%: *p* = 0.27), indicating BMI results were not affected by these characteristics (Figure 2). Although not significantly different, the odds ratios for achieving a BMI reduction of ≥5% or ≥10% with liraglutide versus placebo were smaller for participants with baseline hyperglycemia compared with those with normoglycemia (≥5%: 1.43 vs. 4.36; ≥10%: 1.47 vs. 5.74; Figure 2).

**FIGURE 3:**
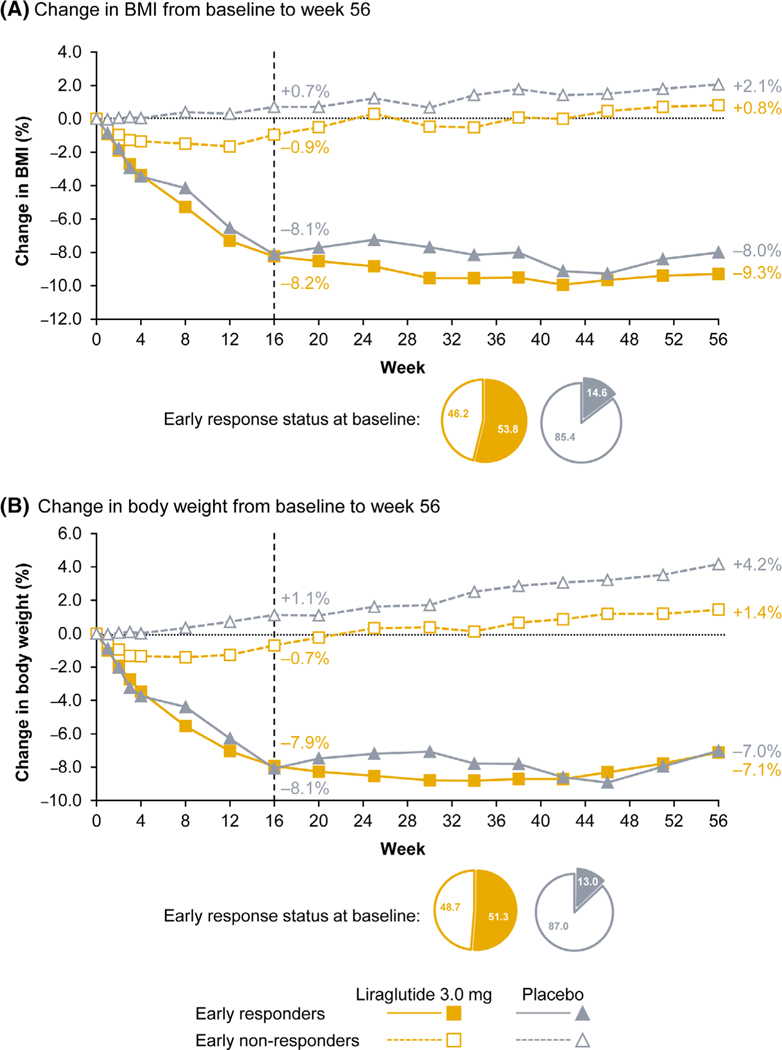
Change in (A) BMI and (B) body weight from baseline to week 56 by early responder status. Data are observed means for participants included in the early responder analysis (i.e., those with baseline and week 16 BMI [Figure 3A] or body weight [Figure 3B] assessments). Each data point is the mean change in BMI (Figure 3A) or body weight (Figure 3B) for all participants with available data at the timepoint. Early responders were participants who achieved ≥4% BMI reduction at week 16 (Figure 3A) or who achieved ≥4% weight loss at week 16 (Figure 3B); early non-responders were participants who did not achieve ≥4% BMI reduction or ≥4% weight loss at week 16. The pie charts present the proportions of participants assessed for early responder status (*n* = 119 for liraglutide, *n* = 123 for placebo) at baseline who were either early responders or early non-responders, as defined by change in BMI (Figure 3A) or body weight (Figure 3B) in each treatment group. BMI, body mass index.

In the liraglutide group, of the 119 participants included in the early responder assessment, 64 (53.8%) were early responders and 55 (46.2%) were early non-responders (Table S1). Among liraglutide early responders with non-missing BMI values at week 56 (*n* = 61), two-thirds (67.2%) achieved ≥5% BMI reductions at week 56, while approximately half achieved ≥10% BMI reductions (49.2%; Table 2). Among liraglutide early non-responders with non-missing BMI values at week 56 (*n* = 49), 16.3% went on to achieve a clinically meaningful BMI reduction of ≥5% at week 56. Early responders to liraglutide who were correctly predicted to achieve ≥5% BMI reduction at week 56 had a mean BMI reduction of 14.0%, while early non-responders to liraglutide who were correctly predicted to not achieve ≥5% BMI reduction had a mean BMI increase of 2.7%. In the placebo group, although the proportion of early responders was low (14.6% of 123 participants included in the analysis [Table S1]), a greater proportion of participants in the early responders group achieved ≥5% and ≥10% BMI reductions at week 56 compared with the early non-responders group (Table S2). Both liraglutide and placebo early responders had greater BMI reductions and weight loss throughout the trial compared with respective early non-responders (Figure 3).

There was no association between weight variability during the 12-week lifestyle therapy-only run-in period and change from baseline in BMI at week 56 for either the liraglutide (*r* = 0.075) or placebo (*r* = −0.089) groups (data not shown).

## 4 | DISCUSSION

Identification of patients most likely to favourably respond to specific AOMs is particularly relevant in the paediatric population in order to minimize unnecessary long-term exposure to pharmacotherapy during development and growth,^7,30^ in addition to enabling efficient use of healthcare resources. This secondary analysis of the SCALE Teens trial aimed to identify possible predictors of liraglutide response in adolescents with obesity with or without T2D. Liraglutide was found to be more effective in reducing BMI by ≥5% and ≥10% compared with placebo regardless of participants’ sex, race, ethnicity, age, Tanner (pubertal) stage, glycemic status, severity of depression symptoms, or obesity category at baseline, indicating that it may be a reasonable treatment option for adolescents with obesity irrespective of these characteristics. In this context, the inclusion of race and ethnicity was a crude surrogate of the structural and societal factors (racism, access to healthcare, poverty, and quality of care) that may predispose someone to developing obesity or being less responsive to AOMs, as race is not a biological marker but rather a social construct.^31^

While none of the baseline characteristics assessed in our study predicted whether adolescent participants achieved ≥5% or ≥10% BMI reduction with liraglutide at week 56, in adults, an exposure-response analysis of three liraglutide trials found that female sex and a lower baseline BMI were associated with greater weight loss compared with male sex and a higher baseline BMI.^32^ The weight loss differences between males and females were partly caused by a higher liraglutide exposure in females compared with males of a similar body weight, while exposure differences attributable to baseline BMI were not associated with meaningful differences in weight loss.^32^ Despite the differences in overall weight loss, participants expected to lose the least amount of weight (males with baseline body weight values in the top 10% of participants) still achieved a clinically meaningful level of weight loss with liraglutide.^32^ In addition, a subgroup analysis of the four adult phase 3 SCALE trials reported a largely similar weight loss treatment difference with liraglutide versus placebo in Hispanic and non-Hispanic participants,^33^ and a retrospective analysis of five randomized clinical trials in adults (including the SCALE trials) found that the effects of liraglutide were consistent across racial subgroups.^34^ Thus, our findings in adolescents appear consistent with those in adults, and show that liraglutide can provide clinically meaningful weight loss regardless of participant characteristics. However, determining whether specific characteristics can affect overall levels of weight loss achieved with liraglutide in adolescents, as sex and baseline BMI have been shown to do in adults, would require further investigation, particularly as body weight is known to affect liraglutide exposure in all age groups.^35^

Some of our findings differ from those previously reported from a small secondary analysis of a clinical trial investigating another GLP-1RA (exenatide 5 μg twice daily) in adolescents.^36^ This exenatide analysis found that, although there was no significant placebo-controlled BMI reduction in males and the treatment effect point estimate was positive (i.e., in favour of placebo), female sex was associated with greater BMI reduction after 3 months of treatment compared with males (treatment effect −4.78% vs. 0.76% in females and males, respectively, *p* = 0.007).^36^ However, similar to the current analysis with liraglutide, baseline BMI and age did not predict the response to exenatide.^36^ The different results found in these analyses may be due to the longer duration and larger population in SCALE Teens compared with this exenatide trial. Although not evaluated in SCALE Teens, results from the exenatide trial demonstrated that higher baseline self-reported appetite was associated with greater BMI reduction.^36^ Thus, eating behaviour phenotypes may represent a potentially promising domain of predictors of response for future paediatric trials of AOMs, as has been demonstrated in adult studies.^37^

In the current analysis, weight variability with lifestyle therapy alone during the run-in period did not appear to affect the ability to lose weight with subsequent liraglutide treatment. This is similar to findings seen in adults with another GLP-1RA (once-weekly subcutaneous semaglutide 2.4 mg), in which weight loss was not affected by either the difference between a participant’s maximum pre-trial body weight and their weight at trial start, or the number of times they had lost ≥5 kg in the past.^38^ These results suggest that an adolescent who meets age and BMI requirements may benefit from pharmacologic treatment regardless of prior weight loss variability, as this does not appear to be a strong predictor of treatment outcome.

Regarding the impact of glycemic status on weight loss, the presence of T2D and prediabetes has been associated with decreased weight loss and maintenance in adults.^39,40^ Similarly, in children, the presence of T2D or prediabetes at baseline has been associated with less weight loss with liraglutide and exenatide treatment compared with those without diabetes or prediabetes.^41^ In our analysis, the presence or absence of T2D or prediabetes at baseline did not significantly affect the degree of BMI reduction with liraglutide versus placebo, although the treatment effect was numerically smaller for participants with baseline hyperglycemia. Interpretation of this finding should take into account the low number of participants with T2D in our analysis.

One of the most consistently identified positive predictors of response to any type of weight loss intervention is an early response to that intervention,^5,14,18–22^ and this appears to also be the case among people treated with liraglutide. In a pooled analysis of data from two SCALE trials of liraglutide for obesity treatment in adults with or without T2D, early responders to liraglutide (defined as achieving ≥4% weight loss at week 16, in line with the FDA liraglutide stopping rule^10^) had greater mean weight loss at week 56 versus early non-responders.^20^ Additionally, greater proportions of early responders (vs. early non-responders) achieved ≥5% and >10% weight loss at week 56 with liraglutide.^20^ We performed a similar analysis using data from SCALE Teens (defining early response as achieving ≥4% BMI reduction at week 16) to assess how the EMA stopping rule^9^ applied to adolescents. Reflective of the adult population, we found early responder status generally provided a strong indication of whether a participant would later achieve a ≥5% BMI reduction with liraglutide. Evaluation of response at week 16 may therefore be of clinical relevance to guide treatment decisions. Of note, 16.3% of liraglutide early non-responders also went on to achieve a clinically meaningful BMI reduction of ≥5% at week 56; in clinical practice, these individuals would have stopped treatment. Given this apparent low probability of going on to achieve a clinically meaningful BMI reduction by week 56, our data suggest it might be more appropriate to consider other treatment options for early non-responders, in line with the stopping rules.

### 4.1 | Limitations

SCALE Teens was not designed to include this secondary analysis and, therefore, not powered to detect differences in treatment effect between the subgroups assessed in this post hoc analysis. As a result, some group sizes were small, such as non-white or Hispanic/Latino participants, those with Tanner (pubertal) stage 2–3, or those with hyperglycemia at baseline, all leading to wider confidence intervals. However, the point estimates suggest that these groups all achieved therapeutic benefit from liraglutide treatment. Further, factors found to be predictors of response for adolescents for other AOMs, such as appetite measures, were not measured in the SCALE Teens trial and, therefore, could not be addressed in this post hoc analysis. Finally, we did not investigate whether adherence differed between subgroups and whether this could have affected our findings. It was previously reported that 81% of liraglutide-treated participants completed treatment in the SCALE Teens trial, indicating good adherence.^11^ However, the proportion of pharmacokinetic samples that had liraglutide concentrations below the lower limit of quantification increased toward the end of the treatment period (6.3% of samples at week 8 vs. 29.6% at week 56), suggesting waning adherence.^11^ Furthermore, the treatment completion rate in SCALE Teens was lower than the 90% of participants who completed treatment with once-weekly subcutaneous semaglutide in the recent STEP TEENS trial in a similar population.^42^

## 5 | CONCLUSION

This secondary analysis found that liraglutide 3.0 mg (or maximum tolerated dose) led to significant reductions in BMI after 56 weeks of treatment regardless of baseline sex, race, ethnicity, age, Tanner (pubertal) stage, glycemic status, obesity category, severity of depression symptoms, and weight variability during the lifestyle therapy-only run-in period. Early response status at 16 weeks was a strong predictor of achieving a clinically meaningful BMI reduction with liraglutide at 56 weeks. These results suggest that liraglutide may be a relevant treatment option for adolescents with obesity with an insufficient response to lifestyle therapy, irrespective of baseline characteristics, and that evaluation of treatment effect after 16 weeks could help guide treatment decisions.

## Supplementary Material

Supplement

## Figures and Tables

**TABLE 1:** Baseline characteristics of all trial participants randomized to liraglutide or placebo.

	Liraglutide (*N* = 125)	Placebo (*N* = 126)
Sex, *n* (%)		
Female	78 (61.9)	78 (61.9)
Race, *n* (%)		
White	105 (84.0)	115 (91.3)
Ethnicity, *n* (%)		
Hispanic/Latino	32 (25.6)	24 (19.0)
Age		
Mean (SD), years	14.6 (1.6)	14.5 (1.6)
Distribution, *n* (%)		
12–14 years	54 (43.2)	64 (50.8)
15–17 years	71 (56.8)	62 (49.2)
Tanner stage,^a^ *n* (%)		
2 or 3	22 (17.6)	21 (16.7)
4 or 5	103 (82.4)	105 (83.3)
Body weight, mean (SD), kg	99.3 (19.7)	102.2 (21.6)
BMI, mean (SD) kg/m^2^	35.3 (5.1)	35.8 (5.7)
Obesity category,^b^ *n* (%)		
Class I	38 (30.4)	39 (31.0)
Class II/III	87 (69.6)	87 (69.0)
Glycemic status, *n* (%)		
Normoglycemia	93 (74.4)	93 (73.8)
Hyperglycemia^c^	32 (25.6)	33 (26.2)
PHQ-9 total score		
Mean (SD)	4 (3)	4 (3)
Distribution, n (%)		
<5	82 (65.6)	85 (67.5)
≥5 to <15^d^	42 (33.6)	41 (32.5)

*Note*: Data for some reported parameters were published in Kelly et al.^11^ Abbreviations: BMI, body mass index; CDC, Centers for Disease Control and Prevention; PHQ-9, Patient Health Questionnaire-9 score; SD, standard deviation.

a For each participant, Tanner stage was the maximum Tanner stage, calculated by combining the results for all categorical questions at the visit. Tanner stage 2 indicates early pubertal development, and stage 5, full maturity.

b Class II/III (severe) obesity (BMI ≥120% of the 95th percentile and/or absolute BMI ≥35 kg/m^2^, whichever was lower)^6^ or Class I obesity (≥95th percentile to <120% of the 95th percentile or absolute BMI <35 kg/m^2^) defined by CDC age- and sex-specific growth charts.^26^

c Prediabetes (fasting plasma glucose 100 to ≤125 mg/dL [5.6 to ≤6.9 mmol/L] or glycated haemoglobin level 5.7% to ≤6.4%) or type 2 diabetes (fasting plasma glucose ≥126 mg/dL [≥7.0 mmol/L] and/or a glycated haemoglobin level ≥6.5%).

d Participants with a PHQ-9 score ≥15 (indicating symptoms of severe depression) at screening or randomization were excluded from the trial.

**TABLE 2:** Positive and negative predictive values for achieving ≥5% and ≥10% BMI reduction with liraglutide 3.0 mg at week 56 by early responder status.

	Liraglutide (*N* = 125)					
	Early responders			Early non-responders		
BMI reduction at week 56	*n* (%)^a^	Positive predictive value,^b^ *n* (%)	Mean BMI change at week 56 for positive predictive value,^c^ %	*n* (%)^a^	Negative Predictive value,^d^ *n* (%)	Mean BMI change at week 56 for negative predictive value,^e^ %
≥5%	61 (55.5)	41 (67.2)	−14.0	49 (44.5)	41 (83.7)	+2.7
≥10%	61 (55.5)	30 (49.2)	−16.5	49 (44.5)	47 (95.9)	+1.5

*Note*: Early responders were participants who achieved ≥4% BMI reduction at week 16; early non-responders were participants who did not achieve ≥4% BMI reduction at week 16. Data are observed (i.e., without imputation).

Abbreviations: BMI, body mass index.

a Proportions are based on the total number of participants included in the early responder analysis (i.e., those with baseline and week 16 BMI assessments) who also had a BMI assessment at week 56 (*n* = 110).

b Early responders who achieved ≥5% or ≥10% (as applicable) BMI reduction at week 56.

c Mean change for early responders who achieved ≥5% or ≥10% (as applicable) BMI reduction at week 56.

d Early non-responders who did not achieve ≥5% or ≥10% (as applicable) BMI reduction at week 56.

e Mean change for early non-responders who did not achieve ≥5% or ≥10% (as applicable) BMI reduction at week 56.
