# Supplementary material for: Evaluating potential predictors of weight loss response to liraglutide in adolescents with obesity: A post hoc analysis of the randomized, placebo-controlled SCALE Teens trial
Source: Pediatr Obes. Author manuscript; Available in PMC 2024 Mar 11. (PMC10926323; doi:10.1111/ijpo.13061)
Supplement: Supplement [file NIHMS1967225-supplement-Supplement.pdf]

SUPPORTING INFORMATION

Evaluating potential predictors of weight loss response to liraglutide in adolescents with obesity: A post-hoc analysis of the randomized, placebo-controlled SCALE Teens trial

Megan O. Bensignor,[1] Carolyn T. Bramante,[1] Eric M. Bomberg,[1] Claudia K. Fox,[1] Paula M. Hale,[2] Aaron S. Kelly,[1] Rashmi Mamadi,[3] Nandana Prabhu,[4] Nina M. Harder-Lauridsen,[5] Amy C. Gross[1]

[1]Department of Pediatrics, Center for Pediatric Obesity Medicine, University of Minnesota, Minneapolis, MN, USA; [2]Clinical Development, Medical & Regulatory Affairs, Novo Nordisk Inc., Plainsboro, NJ, USA; [3]Global Medical Affairs, Novo Nordisk, Bangalore, India; [4]Biostatistics, Novo Nordisk, Bangalore, India; [5]Medical and Science, Novo Nordisk A/S, Søborg, Denmark

Correspondence:

Megan Bensignor

Center for Pediatric Obesity Medicine, Department of Pediatrics, Medical School, University of Minnesota, 717 Delaware ST SE, Minneapolis, MN 55414, USA

Tel: 612-624-5409

Email: moberle@umn.edu

Contents

TABLE S1 Baseline characteristics of all trial participants randomized to liraglutide or placebo according to early responder status 2

TABLE S2 Positive and negative predictive values for achieving ≥5% and ≥10% BMI reduction with placebo at week 56 by early responder status 4

TABLE S1 Baseline characteristics of all trial participants randomized to liraglutide or placebo according to early responder status

	Liraglutide early responders (*N* = 64)	Liraglutide early non-responders (*N* = 55)	Placebo early responders (*N* = 18)	Placebo early non-responders (*N* = 105)
**Sex, *n* (%)**				
**Female**	38 (59.4)	28 (50.9)	10 (55.6)	67 (63.8)
**Age, mean (SD), years**	14.6 (1.6)	14.6 (1.6)	14.7 (1.5)	14.5 (1.6)
**Tanner stage,[a] *n* (%)**				
**2 or 3**	12 (18.8)	10 (18.2)	3 (16.7)	17 (16.2)
**4 or 5**	52 (81.3)	45 (81.8)	15 (83.3)	88 (83.8)
**Body weight, mean (SD), kg**	95.0 (16.8)	103.7 (22.2)	97.2 (16.3)	102.0 (21.0)
**BMI, mean (SD), kg/m^2**	34.1 (3.9)	36.6 (6.1)	34.1 (5.2)	35.9 (5.4)
**BMI SDS, mean (SD)**	3.0 (0.5)	3.3 (0.8)	2.9 (0.7)	3.2 (0.7)
**Glycemic status, *n* (%)**				
**Normoglycemia**	50 (78.1)	37 (67.3)	13 (72.2)	78 (74.3)
**Hyperglycemia[b]**	14 (21.9)	18 (32.7)	5 (27.8)	27 (25.7)

Note: Early responders were participants who achieved ≥4% BMI reduction at week 16; early non-responders were participants who did not achieve ≥4% BMI reduction at week 16. Proportions may not total 100 because of rounding. Data are for participants with baseline and week 16 BMI assessments.

Abbreviations: BMI, body mass index; SD, standard deviation; SDS, standard deviation score.

[a]For each participant, Tanner stage was the maximum Tanner stage, calculated by combining the results for all categorical questions at the visit. Tanner stage 2 indicates early pubertal development, and stage 5, full maturity.

[b]Prediabetes (fasting plasma glucose 100 to ≤125 mg/dL [5.6 to ≤6.9 mmol/L] or glycated hemoglobin level 5.7 to ≤6.4%) or type 2 diabetes (fasting plasma glucose ≥126 mg/dL [≥7.0 mmol/L] and/or a glycated hemoglobin level ≥6.5%).

TABLE S2 Positive and negative predictive values for achieving ≥5% and ≥10% BMI reduction with placebo at week 56 by early responder status

	Placebo (N = 126)					
	Early responders			Early non-responders		
**BMI reduction at week 56**	***n* (%)[a]**	**Positive predictive value,[b] *n* (%)**	**Mean BMI change at week 56 for positive predictive value,[c] %**	***n* (%)[a]**	**Negative predictive value,[d] *n* (%)**	**Mean BMI change at week 56 for negative predictive value,[e] %**
**≥5%**	18 (17.3)	9 (50.0)	–16.8	86 (82.7)	76 (88.4)	+3.8
**≥10%**	18 (17.3)	5 (27.8)	–24.8	86 (82.7)	83 (96.5)	+2.9

Note: Early responders were participants who achieved ≥4% BMI reduction at week 16; early non-responders were participants who did not achieve ≥4% BMI reduction at week 16. Data are observed (i.e., without imputation).

Abbreviations: BMI, body mass index.

[a]Proportions are based on the total number of participants included in the early responder analysis (i.e., those with baseline and week 16 BMI assessments) who also had a BMI measurement at week 56 (*n* = 104).

[b]Early responders who achieved ≥5% or ≥10% (as applicable) BMI reduction at week 56.

[c]Mean change for early responders who achieved ≥5% or ≥10% (as applicable) BMI reduction at week 56.

[d]Early non-responders who did not achieve ≥5% or ≥10% (as applicable) BMI reduction at week 56.

[e]Mean change for early non-responders who did not achieve ≥5% or ≥10% (as applicable) BMI reduction at week 56.
